# Profiles of patients at early stages of Huntington’s disease based on the routine biological markers and the disease progression

**DOI:** 10.1007/s00415-026-13717-0

**Published:** 2026-04-02

**Authors:** Andres Gil-salcedo, Renaud Massart, Katia Youssov, Graça Morgado, Anne-Catherine Bachoud-Levi

**Affiliations:** 1 Département d’Études Cognitives, École Normale Supérieure, PSL University, 75005 Paris, France; 2 Faculté de Médecine, Université Paris-Est Créteil, 94000 Créteil, France; 3 Inserm U955, Institut Mondor de Recherche Biomédicale, Équipe NeuroPsychologie Interventionnelle, 94000 Créteil, France; 4 NeurATRIS, Mondor Node, Créteil, France; 5 Service de Neurologie, Centre National de Référence Maladie de Huntington, APHP, Hôpital Henri Mondor, 94000 Créteil, France; 6 Inserm, Centre d’Investigation Clinique 1430, AP-HP, Hôpital Henri Mondor, Créteil, France; 7 IMRB, Inserm U955, NeuroPsychologie Interventionnelle, Université Paris-Est Créteil, 29 rue d’Ulm, 75005 Paris, France

**Keywords:** Huntinton desease, Biological profiles, Routine Biomarkers, Desease progression

## Abstract

**Background:**

Huntington’s disease (HD) is a neurodegenerative disorder characterized by motor, cognitive, and psychiatric symptoms. Many studies attempt, besides age and CAG, to understand the factors that impact disease progression. Biological tests measuring several metabolic and inflammatory factors are frequently performed in outpatients, but their relation with disease progression is unknown. This study aims to evaluate the association between routine biological profiles at the early manifest stage of HD and subsequent disease progression.

**Methods:**

From 2936 participants, the SPOT-HD database (combination of French part of REGISTRY, BIO-HD and REPAIR-HD), we selected 227 HD mutation carriers at an early stage of HD (HD-ISS stage 2 and CAP < 150), having both longitudinal measurements of routine blood biomarkers and clinical progression assessed using the Unified Huntington’s Disease Rating Scale. Identification of biological routine profiles was performed using an iterative non-parametric model selection with dimensionality reduction algorithm, k-means clustering, LASSO regression, and Random Forest. Disease progression of each profile was estimated using generalized additive modeling.

**Results:**

Three profiles were identified with distinct trajectories of HD progression (*p* < 0.001). The Rapid-Progression Profile with relatively higher levels of triglycerides, liver enzymes, and body mass index, coupled with lower high-density lipoprotein (HDL) cholesterol levels was noted. The Intermediate-Progression profile with relatively higher HDL levels, lower triglycerides, and inflammatory and liver markers in the lower end of the normal range was noticed. The Slow-Progression Profile with a tendency for higher T4 and creatinine levels, a greater concentration of lymphocytes than in other profiles, and low HDL despite normal triglycerides were studied.

**Conclusion:**

Biological routine profiles may help anticipating HD progression, thus facilitating personalized medicine approaches. These findings suggest that routine metabolic and inflammatory markers may be useful for patient follow-up in HD.

**Supplementary Information:**

The online version contains supplementary material available at 10.1007/s00415-026-13717-0.

## Introduction

Huntington’s disease (HD) is an autosomal dominant neurodegenerative disorder characterized by a triad of motor, cognitive, and psychiatric symptoms [1]. Despite the strong association between age and the number of cytosine–adenine–guanine (CAG) repeats with disease progression, HD displays considerable heterogeneity in the onset of symptoms, clinical manifestations, and rate of progression [2]. This clinical variability suggests that factors beyond the primary genetic mutation influence the disease trajectory. Emerging evidence indicates that systemic processes, including metabolic dysregulation [3], chronic inflammation [3, 4], and cardiovascular comorbidities [3], are associated with accelerated neurodegeneration in HD although the underlying biological pathways remain unclear [1, 5]. This observed heterogeneity complicates the development of reliable prognostic models and effective therapeutic strategies. It underscores the need for a more personalized approach that considers the complex interplay between genetic modifiers, environmental influences, and systemic complications [6, 7].

Efforts to identify potential profiles in HD patients have largely relied on non-routine biomarkers, such as neuro-filament light chains (Nfl) [8–10], m-RNA, mutant huntingtin protein quantification [11], and other complex biomarkers in combination with advance neuroimaging [12]. While these tools provide detailed insights, their limited accessibility due to high cost, technical complexity, and lack of standardization limits their usefulness in daily clinical practice at large-scale [2, 10]. This calls for affordable and reproducible biomarkers reflecting the health profiles of HD mutation carrier participants. One approach is to utilize routine biomarkers commonly collected in general clinical settings, such as lipid profiles, glycemic indices, body mass index, inflammatory markers, and liver enzymes, which may be associated with HD progression [13, 14].

Among routine biomarkers, altered lipid metabolism, including changes in cholesterol and lipoprotein composition, has been observed in both pre-manifest and manifest HD patients [15–17]. Specifically, studies have indicated that HD patients exhibit reduced levels of cholesterol precursors, such as lathosterol and lanosterol, in both plasma and brain tissues [18, 19]. Systemic lipid dysregulation appears to worsen over time, characterized by altered fatty acid metabolism and elevated markers of lipid peroxidation [20]. In pre-manifest HD (HD-ISS stages 0 and 1) [21], plasma cholesterol and low-density lipoprotein (LDL) components are already diminished, suggesting that metabolic disruption may precedes clinical symptoms [15, 19]. Parallel to lipid abnormalities, impaired glucose metabolism disrupts energy homeostasis, increasing neuronal vulnerability and contributing to neurodegeneration in HD [4, 22, 23]. Hyperglycemia and insulin resistance further exacerbate metabolic stress and oxidative damage, accelerating disease progression and motor decline [22, 24]. Additionally, elevated inflammatory markers (e.g., lymphocyte, monocyte) and liver function markers (e.g., alkaline phosphatase, gamma-glutamyl transferase) may be associated to an earlier onset age, and a faster disease progression [25–27] and neurodegeneration via metabolic dysfunction and oxidative stress [15, 18, 28, 29]. These findings highlight that routine biomarkers not only reflect metabolic and inflammatory states but they may also provide insights into the interactions between HD progression and comorbidities, such as diabetes, dyslipidemia, and cardiovascular disease, increasingly recognized as modifiers of neurodegenerative disease course [13, 14].

The integration of routine biomarkers in HD profiles could address critical challenges: First, it has the potential to reduce heterogeneity and enhancing statistical power in therapeutic trials by helping stratification [14, 30]. Second, it may provide clinicians with useful tools to prognosticate disease progression and exacerbations related to comorbidities across various care settings [30]. Identifying progression profiles during the early stages of the disease, before irreversible functional deterioration, may prompt therapeutic interventions aimed at delaying disability or mitigating symptom severity [14, 30].

Rather than analyzing routine biological markers in isolation, one promising strategy to identify patient profiles is to use a clustering approach that groups patients with similar biological patterns, which may reflect shared underlying pathophysiological processes. This method may reveal combinations of markers that, together, are associated with various disease progression, even when individual biomarker values fall within the normal clinical range. However, the link between these eventual combinations and the progression of HD remain unclear [13, 14, 30]. Therefore, this study aims to evaluate the association between routine marker-based profiles at HD-ISS stage 2 and disease progression.

## Methods

### Study population

The data for this study were extracted from the SPOT-HD database, which is the combination of the French-Creteil data of REGISTRY (NCT01590589), BIO-HD (NCT01412125), and REPAIR-HD (NCT03119246) databases. All these studies were conducted at or under the supervision of the National HD Reference Center at the Henri Mondor Hospital Neurology Department. The three databases under consideration share a standard data collection protocol and a substantial number of participants diagnosed with HD. This similarity allows the reconstruction of the clinical course of these participants up to 22.7 years after their enrollment (mean = 3.82, SD = 3.88). REGISTRY is an observational study of the European Huntington’s Disease Network (EHDN) that systematically collected genotypic, phenotypic, demographic, and biomarker clinical data from HD manifest and pre-manifest mutation carriers and controls. BIO-HD and REPAIR-HD are two observational studies designed for the identification of HD-associated biomarkers and the development of assessment tools for HD follow-up.

We selected participants older than 18 years with a confirmed diagnosis of HD mutation of 40 or more CAG repeats, at early phase of the disease, with at least two visits, who are the target of future interventions. We thus selected participants at HD Integrated Staging System (HD-ISS) [31] stage 2 (no functional disability but motor or/and cognitive symptoms) and the standardized CAG-age-product (CAP) [32] score of less than 150 (for which 100 is the expected age at diagnosis). Participants who did not have routine biological markers data were excluded from the analysis.

### Disease progression, analysis inclusion visits, and follow-up

We used the Composite Unified Huntington’s Disease Rating Scale (cUHDRS) to provide a global evaluation of the clinical status of Huntington’s disease (HD) gene carriers with varying degrees of functional impairment [33]. The cUHDRS integrates functional (Total Functional Capacity [TFC]), motor (Total Motor Score [TMS]), and cognitive (Symbolic Digital Modalities Test [SDMT], and Stroop Word Reading [SWR]) measures. We used the first cUHDRS assessment performed when individuals with a CAP score below 150 entered HD-ISS stage 2 as a baseline to construct standardized marker-based profiles. All subsequent cUHDRS assessments were included until the end of follow-up to characterize the disease trajectory.

### Routine markers

A blood sample was collected from each participant at every visit. Routine health markers were analyzed. While morning collection was generally recommended, a strict fasting state was not mandated. The extracted blood markers included: electrolytes (sodium, potassium, and chloride levels); endocrine markers (triiodothyronine [T3] and thyroxine [T4]); hematological factors (red blood cells [RBC], hemoglobin, and platelets); hepatic markers (alanine aminotransferase [ALT], gamma-glutamyl transferase [GGT], total bilirubin, alkaline phosphatase [ALP], and aspartate aminotransferase [AST]); inflammatory/immunological markers (white blood cells [WBC], neutrophils, lymphocytes, and monocytes); metabolic markers (triglycerides, high-density lipoprotein [HDL], total cholesterol, blood glucose, and low-density lipoprotein [LDL]); and renal markers (creatinine and urea). We focused on markers previously associated with HD or its progression in previous studies [34]. In addition, data from patients with more than 60% missing markers data were excluded to ensure robustness.

During the same visit, participants' weight and height were measured to calculate their body mass index (BMI). Weight data were imputed for 32% of the total observations due to the low variability in weight among participants before functional deterioration onset (see supplementary material and methods section). Additionally, socio-demographic data (age, sex, and years of education), health behaviors (tobacco, alcohol, or drug use), and information on exposure to various medications including antidepressants, anxiolytics, antipsychotics, neurological, cardiovascular, antidiabetic, lipid-lowering, endocrine, and cancer treatments were collected.

### Statistical analysis

An exploratory analysis was performed of the specific association of each biomarker with disease progression, using a linear mixed model adjusted for age, CAG and their interactions. The estimation of routine marker-based profiles was carried out through a sequential variable selection process using baseline visit data. The process for selection and identification of profiles involved several steps. First, we standardized the markers using *Z*-scores and applied a dimensionality reduction technique (Uniform Manifold Approximation and Projection, or UMAP) to transform the high-dimensional biomarker values into a simpler representation, facilitating the identification of patterns. Using these simplified data, we then applied a k-means clustering algorithm to identify groups of participants with similar biomarker profiles. The optimal number of clusters was determined by evaluating which grouping showed the maximal differences in cUHDRS trajectories. Trajectories were modeled using a Generalized Additive Model (GAM), which can flexibly capture non-linear effects.

To select the key biomarkers defining these profiles, two complementary approaches were used: LASSO (least absolute shrinkage and selection operator) regression, a method that eliminates the least informative variables, and a Random Forest model, which assesses the relative contribution of each marker. With each iteration, as the number of biomarkers decreased, the number of participants without missing data increased. This entire procedure was repeated until a final, stable set of key biomarkers was identified (see supplemental statistical methods for more details).

A final GAM model was then calibrated with the final set of identified markers, adjusted for socio-demographic factors and health behaviors. This model also assessed the mediating effect of each of the biomarkers on the association between the identified profiles and their association with the cUHDRS trajectory. All analysis was carried out in R version 4.2.1 (2022-06-23 ucrt).

### Sensitivity analysis

Given the multidimensional character of the cUHDRS, GAM models were calibrated for TMS, SDMT, STW, and TFC to avoid an inconsistent impact of the profiles on each clinical outcome. To mitigate the possibility of overfitting in the non-linear GAM model calibration, a linear mixed model (more restrictive) was also calibrated to evaluate the trajectories of each profile. To evaluate the impact of data imputation on the stability of our findings, we replicate the final model using only the subset of participants with non-imputed BMI data.

### Standard protocol approvals, registrations, and patient consents

The studies were all approved by Ile de France: Henry Mondor Hospital (Creteil) et Saint Louis Hospital (Paris), French Ethical Committees (CPP). Further, these were conducted in accordance with the local legislation and institutional requirements. Written informed consent was obtained from the participants or the participants' legal guardians/next of kin in accordance with the national legislation and institutional requirements.

## Results

**Fig. 1:**
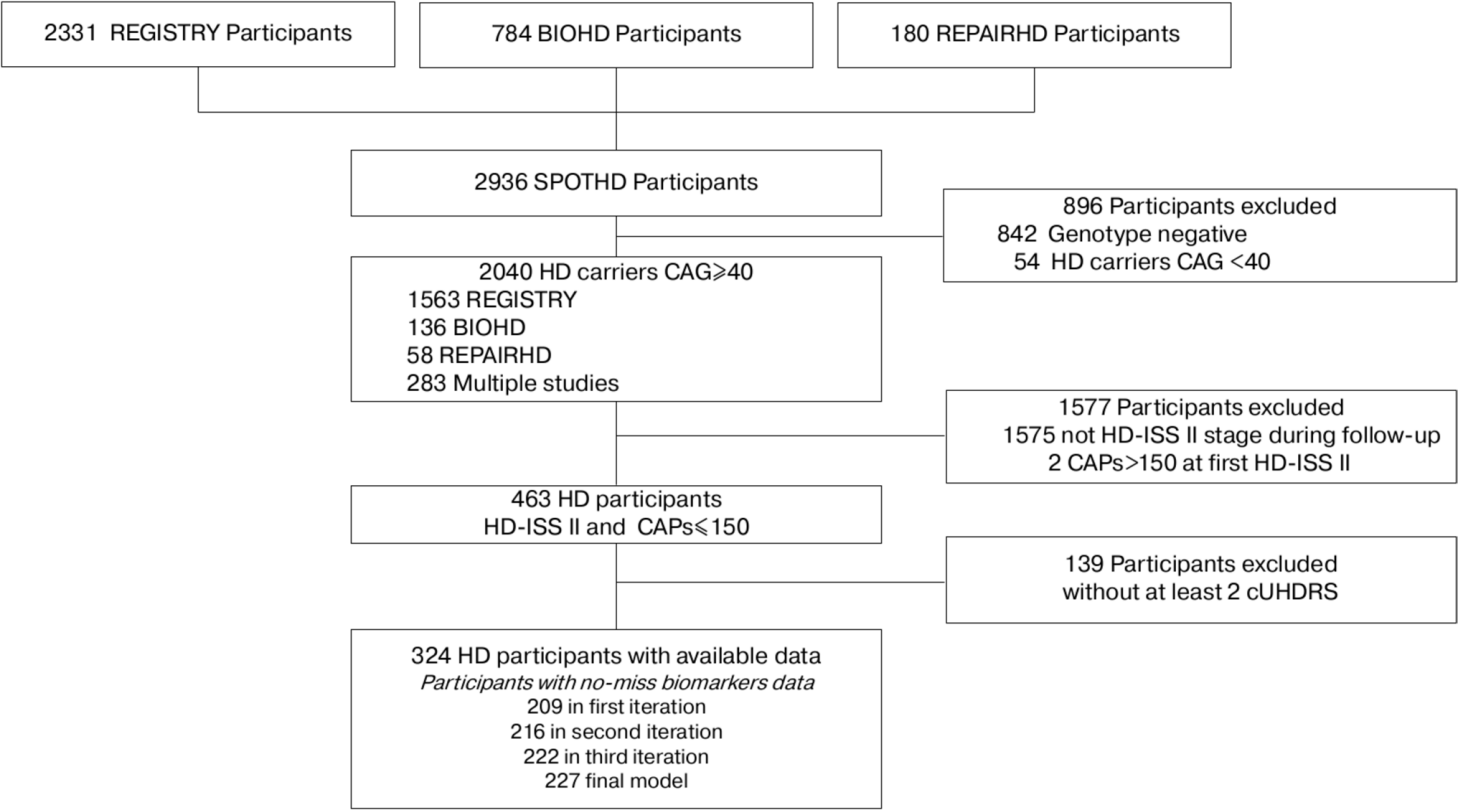
Flowchart of Participant Selection from the SPOT-HD Database. *HD* Huntington’s disease, *CAPs* CAG-age-product standardized, *CAG* cytosine–adenine–guanine, *cUHDRS* Composite Unified Huntington’s Disease Rating Scale

The SPOT-HD database comprised 2331 participants from REGISTRY, 784 from BIO-HD and 180 from REPAIR-HD (359 individuals co-enrolled, Fig. 1). Of the 2936 total SPOT-HD participants, 896 genotype negative participants (CAG repeats < 36) and HD carriers with less than 40 CAG repeats were excluded. In addition, 1577 participants lacking at least one observation with an HD-ISS stage 2 with CAPs < 150 were excluded. Finally, 139 participants were excluded due to a lack of at least two complete clinical evaluations. This yielded a total of 324 participants with available routine marker data, who were retained for the marker selection phase.

The socio-demographic and clinical characteristics of participants with available data are described in Table 1. With respect to blood markers and BMI, biomarkers values remained within the normal range for most of them, although some notable deviations were observed (see Figure S1). Triglycerides (mean = 1.00 [0.57]), liver enzymes (ALAT = 21.60 [9.85], ASAT = 21.21 [5.87], GGT = 22.89 [14.07]), and bilirubin (mean = 9.63 [4.91]) exhibited a skewed distribution, which may suggest metabolic or hepatic alterations in several individuals. The lipid profile showed a large variability in total (5.34 [1.02]) and LDL cholesterol (mean = 3.18 [0.89]), indicating the potential for cardiovascular risk in some participants. Hematologic and electrolyte markers were stable. We observed an association between LDL, HDL, T3, WBC, and urea levels, and cUHDRS progression; however, this association was not maintained following p value adjustment (Table S1).

**Table 1:** Population characteristics for the routine biomarker selection phase at the first cUHDRS measurement in HD-ISS stage 2 and CAP < 150

Characteristics	Overall (*N* = 324)
Age (years)	47.69 (12.41)
Sex	
Male	170 (52.5%)
Female	154 (47.5%)
Years of education	12.17 (3.52)
CAG repeats	44.02 (3.07)
CAG-age-product standardized	98.80 (16.90)
Total motor score	20.84 (14.88)
Symbol digit modalities test	32.02 (13.36)
Stroop word test	73.54 (18.54)
Total functional score	12.22 (1.50)
cUHDRS	12.24 (3.15)
Alcohol consumption	
No	303 (93.5%)
Yes	21 (6.5%)
Tobacco consumption	
No	227 (70.1%)
Yes	97 (29.9%)

Mean (standard deviation) except where otherwise indicated

*cUHDRS* Composite Unified Huntington’s Disease Rating Scale, *CAG* cytosine–adenine–guanine

The biomarker selection process was ran in a total of three iterations (see Supplemental Results and Fig. 1 and S2), followed by the final model. While 324 participants in HD-ISS stage 2 initially had biological data available, an iterative selection process was performed to optimize data completeness. By refining the biological panel and excluding biomarkers with low predictive value or high missingness (such as sodium, potassium, bilirubin, and chloride), the sample size for complete-case analysis increased. This iterative approach (detailed in Supplementary Results) allowed the final progression model to be calibrated with 227 participants who possessed a complete set of the optimized biomarkers and at least two clinical evaluations.

**Fig. 2:**
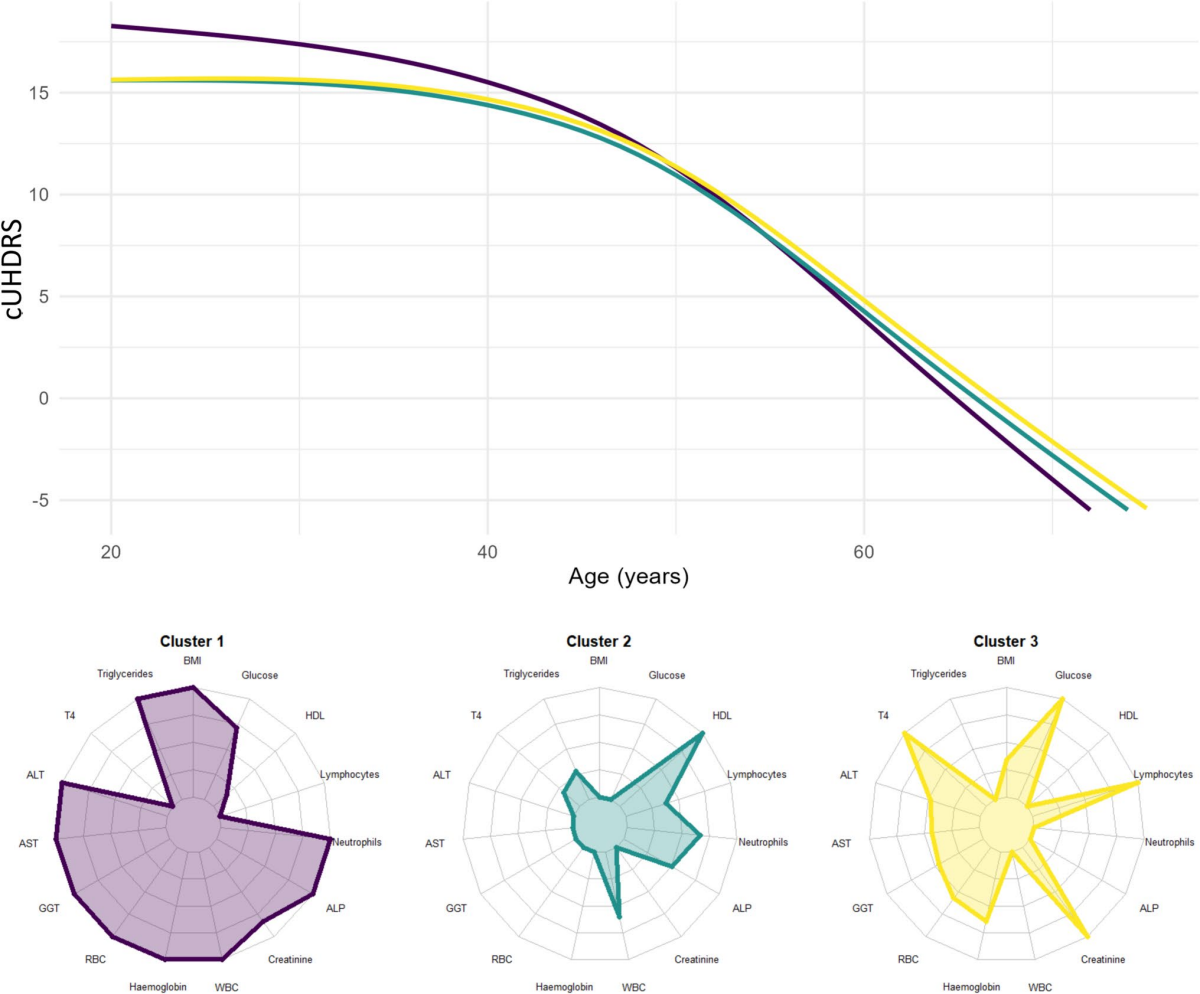
Trajectories of HD progression measured with the cUHDRS as a function of retained -set-based profiles of routine markers in individuals at HD-ISS stage 2. Trajectories of model presented in Table 2. *T4* thyroxine, *RBC* red blood cells, *ALT* alanine aminotransferase, *GGT* gamma-glutamyl transferase, *ALP* alkaline phosphatase, *AST* aspartate aminotransferase, *WBC* white blood cells, *HDL* high-density lipoprotein

The final set of retained routine biomarkers consisted of 15 markers, based on which three clusters were identified, grouping 95, 90, and 42 participants, respectively, with significantly different metabolic and hematologic profiles and HD progression trajectories (Figs. 2 and S3). At baseline, age, years of education, CAG repeats, tobacco and alcohol consumption, and clinical scores (TMS, STW, SDMT, TFC, cUHDRS) were similar across the three clusters (Table S2). A higher proportion of women was observed in Cluster 2 (Table S2) compared to the two other clusters (*p* < 0.001). Clusters differed also significantly in triglyceride, T4, glucose, and HDL levels, as well as in the remaining biomarkers (*p* < 0.0001 for all, Fig. S4). Specifically, Cluster 1 (depicted in purple) exhibited the highest triglyceride levels, while Cluster 2 (depicted in green) demonstrated the highest HDL and the lowest glucose levels (4.79 [0.47] mmol/L). In Cluster 3 (depicted in yellow) although higher mean glucose levels were observed, these did not differ significantly from those in Cluster 2 (5.35 [0.45] vs. 5.19 [0.65], *p* > 0.755). Liver enzymes, including ALT, AST, and GGT, exhibited differential values across the clusters; they were elevated in Cluster 1 compared to the other two clusters. Levels of hemoglobin and RBC were higher in Cluster 1 while lymphocytes and neutrophils were higher in Cluster 3. Additionally, creatinine level was the highest in Cluster 1.

The final GAM, fully adjusted, showed that the routine marker-based profiles (clusters) were significantly associated with cUHDRS trajectories with age as the time scale (Deviance explained = 80.5%, Fig. 2). Cluster 1 showed a higher intercept score, followed by Cluster 2 and Cluster 3 (Table 2). However, individuals from all clusters declined with age, with Cluster 1 showing the most pronounced slope (*β* = −0.78; 95%CI −0.90 to −0.66; *p* < 0.001) with an acceleration of the decline appearing from around 39 years. Cluster 2 exhibited the second fastest decline (*β* = −0.67; 95%CI −0.80 to −0.55; *p* < 0.001), with an acceleration around 42 years old. Conversely, Cluster 3 exhibited the slowest decline (*β* = –0.66, 95%CI −0.78 to −0.55, *p* < 0.001), with a subsequent acceleration occurring around 45 years old. Education was associated with a better cUHDRS score at baseline (*β* = 0.91; 95% CI 0.37–1.43; *p* = 0.001).

**Table 2:** Regression model results for the estimation of cUHDRS according to retained-set-based profiles of routine biomarkers in individuals at HD-ISS stage 2

	Full adjusted model			Final model retained		
Variable	Estimates	CI	*p*	Estimates	CI	*p*
Cluster 1 purple	**30.61**	**26.34–34.88**	** < 0.001**	**30.47**	**26.78–34.15**	** < 0.001**
Cluster 2 green	27.01	21.98–32.03	< 0.001	26.74	22.96–30.52	< 0.001
Cluster 3 yellow	26.93	22.35–31.52	< 0.001	26.77	22.77–30.77	< 0.001
Cluster 1 purple*age	−0.78	−0.90 to −0.66	< 0.001	−0.78	−0.90 to −0.66	< 0.001
Cluster 2 green*age	−0.68	−0.80 to −0.55	< 0.001	−0.67	−0.80 to −0.55	< 0.001
Cluster 3 yellow*age	−0.66	−0.78 to −0.53	< 0.001	−0.66	−0.78 to −0.53	< 0.001
CAG repeats	−6.94	−7.88 to −6.00	< 0.001	−6.96	−7.89 to −6.03	< 0.001
Years of education	0.91	0.37–1.44	0.001	0.9	0.37–1.43	0.001
Smooth term (age)	–	–	< 0.001	–	–	< 0.001
ti (Age*CAG repeats)	–	–	< 0.001	–	–	< 0.001
Smooth term (participant)	–	–	< 0.001	–	–	< 0.001
Alcohol consumption (0 = non)	0.36	−0.38–1.10	0.340			
Tobacco consumption (0 = non)	−0.26	−1.09–0.56	0.532			
Sex (0 = male)	−0.09	−1.80–1.62	0.916			

GAM estimates considering the influence of metabolic profiles (clusters) on cUHDRS progression. Full adjusted model includes all explored variables. Final model retained presents the final version with only significant variables. Parametric terms were used for the age, cluster, and their interaction. Smooth term was applied to model longitudinal effect of age. A tensor product smooth term (ti) was applied to model the interaction between age and the number of CAG repeats. Additional smooth terms were included for age and between-individual variability (participant) as random effect

*cUHDRS* Composite Unified Huntington’s Disease Rating Scale, *CI* confidence interval, *CAG* Cytosine–adenine–guanine repeats in the HTT gene

None of the singular marker influenced substantially the association between the profiles and the cUHDRS trajectory in the mediation analysis (Table S3). The most relevant, yet modest, influences on the intercept were observed separately to be BMI, HDL, ALP, and blood glucose separately (percentage mediated 3–8%), with BMI being the most prominent. Conversely, the percentage of mediation in the decline of each cluster according to age differed from 0% only for ALP and HDL (1–2%).

### Sensitivity results

GAM models adjustments for age, CAG, interactions between age and CAG, years of education, and a random effect per participant for the TMS, SDMT, STW, and TFC yielded results consistent with the primary findings (Fig S5). The linear mixed model, despite failing to demonstrate a statistically significant difference between Cluster 2 and Cluster 3, exhibited similar trajectories to the mean results (Fig S6). To evaluate the impact of data imputation on the stability of our findings, a sensitivity analysis was performed using only the subset of participants with non-imputed BMI data. The model estimated solely with this non-imputed dataset yielded results consistent with the primary analysis, identifying similar cluster profiles and longitudinal progression trajectories (Supplementary Fig S7). Finally, the model estimated solely with this non-imputed dataset yielded results consistent with the primary analysis (Fig S7).

## Discussion

This longitudinal study, which included more than 227 participants with individuals with Huntington’s disease without functional limitation (namely at HD-ISS stage 2), identified three distinct progression profiles based on a final set of 15 routine biomarkers. Each profile displayed a unique biological pattern with mean values for most biomarkers within normal clinical range (Figure S1) but in the unique combination and relative distribution of these markers. These patterns define profiles associated with significantly different rates of disease progression.

The Rapid-Progression Profile (Cluster 1) was distinguished by a trend toward relatively higher levels of triglycerides, liver enzymes (ALT, AST), and BMI, coupled with lower HDL cholesterol levels compared to the other groups. Although most patients in this cluster did not meet the diagnostic criteria for dyslipidemia or liver disease, this pattern suggests a state of subclinical "metabolic–inflammatory stress" that may accelerate HD progression. The Intermediate-Progression Profile (Cluster 2) could be considered a low-risk metabolic profile. These patients showed, on average, relatively higher HDL levels, lower triglycerides, and inflammatory (WBC) and liver markers in the lower end of the normal range compared to Cluster 1. The Slow-Progression Profile (Cluster 3) presented the most complex pattern, with a tendency for higher T4 and creatinine levels, a greater proportion of lymphocytes (within a lower total WBC count), and a distinctive lipid profile of low HDL despite normal triglycerides. This "hematologic–thyroid" profile was associated with the slowest disease progression, suggesting that certain metabolic and immune interactions could have a modulatory or even compensatory effect in HD mutation carriers. The three profiles showed a robust association with no predominant influence by any specific biomarker. However, BMI, ALP, HDL, and blood glucose were shown to be those with slight but relevant mediation.

Our findings for Cluster 1 align with evidence that suggest that metabolic dysfunction and systemic inflammation are associated with accelerated HD progression [23, 25, 27]. Although the levels of lipids and liver enzymes in our cohort were not abnormal, the observed pattern of relatively higher triglycerides and lower HDL is consistent with prior studies, which have reported alterations in lipid metabolism in HD mutation carriers, including reduced HDL and LDL sub-fractions (e.g., HDL4 and LDL3–6) [15, 17] and elevated VLDL5 components, suggesting a localized dysregulation in triglyceride metabolism [15, 17, 19]. Whereas these lipidic alterations correlate with motor decline and striatal atrophy [16, 35], direct evidence associating hypertriglyceridemia to neurodegeneration remains limited in general literature. While deficits in cholesterol biosynthesis indicate a possible hepatic metabolic dysfunction [18], further studies are needed to clarify the extent of liver dysfunction in HD [29, 36]. Moreover, the association between elevated levels of transaminases (ALT/ASAT) and the progression of HD [36] is consistent with preclinical (animal models and in vivo studies) and clinical (pilot observational study) findings that suggest hepatic mitochondrial dysfunction contribute to neurodegeneration through systemic oxidative stress [28, 37].

The association of Cluster 3 with slower progression requires careful interpretation. This "hematologic–thyroid" profile confirms that the associations between immune, endocrine, and metabolic systems is relevant in HD [38, 39]. Our results may contrast with those of previous studies in which a higher T4 level was associated with a larger size of mutant CAG repeats, which normally correlate with faster disease progression [38]. However, individuals in our Cluster 3 exhibited mean T4 levels in the high-normal range (mean = 14.96 [3.07]). Presumably, T4 levels in this upper range (with thyroid hormone receptor α) may promote protective mechanisms, such as autophagy and neuronal mitochondrial function [40], processes known to be altered in HD [41, 42]. Therefore, the effect of T4 might be context-dependent, influenced by its interaction with other factors, such as immune status and muscle mass (as suggested by the relatively higher creatinine levels in this group). Indeed, elevated creatinine may indicate preserved muscle mass, in conjunction with non-obese overweight. It could act as a metabolic reserve, mitigating cerebral energy catabolism [43, 44]. In addition, this profile showed increased lymphocytes (mean = 33.66%) despite a lower total WBC count (mean = 5.31 [1.21]). This may suggest a shift in leukocyte composition rather than overt inflammation or immunosuppression [25]. The combination of low HDL, normal triglycerides, and elevated T4 suggests an altered lipid redistribution that could mitigate oxidative stress [20, 45, 46], highlighting the complex interplay between endocrine, immune, and metabolic systems in HD progression [20, 45]. Finally, previous studies suggest that stable, low-inflammatory metabolic profiles (Cluster 2) may modulate HD progression by influencing cellular energy balance [47, 48], mitochondrial function [47] and neuroinflammation [49].

Therefore, routine biomarker use may help clinical management and the design of therapeutic trials. The identification of the Cluster 'metabolic–inflammatory' profile (Cluster 1) indicates a high-risk subgroup where the association of dyslipidemia, central adiposity, and systemic inflammation with rapid progression warrants further investigation into whether metabolic management (e.g., statins, dietary modifications) could slow progression [50]. The association between 'haematological–thyroid' profile (Cluster 3) and slow progression gives rise to the potential exploration of immunomodulation strategies (e.g., therapies that boost regulatory lymphocytes) or controlled thyroid supplementation. Furthermore, these findings underline the need to investigate cross-mechanisms between peripheral systems and neurodegeneration, and to consider the interaction between different markers and assess them together.

The strengths of our study were the robust sample size at HD-ISS stage 2, the relatively large number of routine biomarkers available and the persistence of the results after models adjustment for genetic and socio-demographic factors of a multivariate analysis. It has the interest to replace in the framework of HD progression routinely collected data usually collected for searching comorbidities. Yet this does not allow to establish any causality between profiles and progression. In addition, pharmacological treatments were only available for 60 participants of analyzed population, which precluded assessing their influence on the trajectories of each profile. Additional easily accessible markers but beyond routine were not measured (e.g., specific immunological markers like lymphocyte subpopulations, IL-6) that could explain the possible lymphocytosis. Elevated creatinine levels, while indicative of preserved muscle mass, were not confirmed by more direct measures, such as bioelectrical impedance or computed tomography. Blood collection was not strictly standardized for fasting state, which may influence certain markers like triglycerides and glucose.

A further limitation is that our analyses were restricted to individuals classified as HD-ISS stage 2. While this focus allowed for robust and clinically meaningful modeling of early manifest disease trajectories, participants in pre-manifest (HD-ISS stages 0–1) and more advanced stages were underrepresented in the available cohorts, precluding statistically reliable analyses outside stage 2. However, HD-ISS stage 2 represents a relatively homogeneous clinical phase in which subtle but measurable changes are already detectable, making it particularly well suited for evaluating biomarker-associated differences in early disease progression and their relationship to emerging clinical manifestations. Furthermore, the generalization of the results requires replication in multicultural cohorts in HD-ISS stages 0 and 1. In addition, the lack of longitudinal biomarker data limits the assessment of their temporal dynamics. The relatively low mean TFC score observed in our HD-ISS stage 2 cohort reflects the fact that HD-ISS classification does not rely on the traditional clinical interpretation of TFC (i.e., considering a score of 13 as universally normal), but rather on a statistical probability model that accounts for age. Therefore, a TFC score of 8–9 may still fall within the expected normative range for an older individual (e.g., 90 years old).

This study relates possible metabolic, inflammatory, and hematological comorbidities to disease heterogeneity. It shows the potential utility of accessible and cost-effective tools for personalized medicine strategies in HD, thus highlighting that the management of systemic comorbidities could complement approaches targeting the central pathology.

## Supplementary Information

Below is the link to the electronic supplementary material.

Supplementary file1 (PDF 2218 KB)

## Data Availability

The datasets generated during and/or analyzed during the current study are not publicly available due to sensitive patient information but are available from the corresponding author on reasonable request.
