# Supplementary material for: Profiles of patients at early stages of Huntington’s disease based on the routine biological markers and the disease progression
Source: J Neurol. 2026 Apr 2;273(4):249. doi: 10.1007/s00415-026-13717-0 (PMC13046660; doi:10.1007/s00415-026-13717-0)
Supplement: Supplementary file 1 — Supplementary file1 (PDF 2218 KB) [file 415_2026_13717_MOESM1_ESM.pdf]

Supplementary Methods

Supplemental methods.

Of the participants included in the analysis, 32% exhibited missing weight data during at least one visit, and imputation was performed using Chained Equations methodology, with consideration given to the longitudinal nature of the data. The imputation model was fitted with the following variables: age, sex, years of education, alcohol consumption, drug use, tobacco use, height, high- and low-density cholesterol, total cholesterol, triglycerides and glycaemia, as well as total motor score, total functional score, symbol digital test, Stroop Word test, cUHDRS and the independence scale. Each subject was added as a random effect. The imputation was conducted using the mice package in R.

Supplemental statistical methods.

For the main statistical analysis, we employed a stepwise procedure to derive participant profiles based on routine blood biomarkers collected at baseline. This multistage approach integrated dimensionality reduction, unsupervised clustering, and variable selection to identify biologically meaningful subgroups associated with differences in clinical progression.

1. First, all biomarker values were standardized to ensure comparability across variables. We then applied Uniform Manifold Approximation and Projection (UMAP), a non-linear dimensionality reduction technique that captures both local and global relationships in the data. This allowed the projection of the high-dimensional biomarker space into two principal components, preserving structure while enabling the identification of participant-level patterns.
2. The resulting UMAP components were used as input for k-means clustering to group individuals with similar biomarker profiles. To determine the optimal number of clusters, we systematically evaluated solutions ranging from 2 to 5 clusters. For each

solution, we compared the longitudinal clinical trajectories across groups using Generalized Additive Models (GAMs) fitted on the Composite Unified Huntington's Disease Rating Scale (cUHDRS). Model comparisons were based on the explained deviance (for non-normal distributions) and the statistical significance (p-values) of the differences in cUHDRS trajectories between clusters. The clustering solution that maximized the difference in progression while maintaining interpretability and cluster stability was selected for further analysis.

3. Disease progression was modelled using GAMs with a scaled t-distribution to account for potential outliers and non-Gaussian errors. Age was modelled using a cubic regression spline with six basis functions to capture non-linear effects. Additionally, a tensor product spline term modelled the interaction between age and the number of CAG repeats, accounting for their combined influence on disease trajectory. A subject-specific random effect was also included to adjust for within-individual correlation in repeated measurements.
4. To identify the most informative biomarkers contributing to the cluster structure and progression differences, we used two complementary feature selection methods. First, we implemented logistic or multinomial regression with a Least Absolute Shrinkage and Selection Operator (LASSO) penalty, which forces non-informative variables' coefficients to zero. Second, we trained a Random Forest classifier that included an artificial cut-off variable composed of random values. The mean decrease in Gini index attributed to this variable served as a benchmark: biomarkers with an equal or lower contribution than the cut-off variable were excluded. Only biomarkers retained by both methods were selected for the next iteration.

5. This selection procedure was repeated iteratively until all retained biomarkers consistently had non-zero coefficients in the LASSO model and exceeded the Gini importance threshold in the Random Forest analysis.

Finally, a GAM model was calibrated with the final set of biomarkers, adjusted for sociodemographic variables and health-related behaviours. This final model also included mediation analyses to estimate the extent to which each biomarker mediated the association between biomarker-derived profiles and clinical progression measured by cUHDRS.

Supplemental Results.

The biomarker selection process was executed in a total of three iterations (see Figure S2), and the final analysis. The initial iteration was estimated with a total of 26 biomarkers and 209 participants with data for all biomarkers at baseline (Figure S2, Panel A). The GAM-1 model, with a deviance (due to non-linear distributions) explained of 80.3%, identified two clusters with significantly different trajectories of cUHDRS. Eight of the biomarkers (sodium, monocytes, platelets, potassium, LDL, TSH, T3, and total cholesterol) that did not show to be determinant in cluster classification in the LASSO logistic regression model and were below the cut-off variable in the mean Gini reduction of the random forest, were excluded. In second iteration with 216 participants, the GAM-2 model, which explains 80.5% of the deviance, identified three clusters with significantly different trajectories (Figure S2, Panel B). Cluster number 3 showing a significantly faster decline than the other two. The LASSO multinomial regression and the cut-off method in the estimation of the mean Gini reduction of the random forest determined that total bilirubin and urea were not included in the estimation of the clusters, and thus, they were excluded. In the third iteration, three clusters were estimated with 222 participants in the last iteration with a GAM-3 model that explained 80.7% of the deviance. The biomarker chloride was excluded due to its mean Gini reduction lower than the cut-off

variable in the random forest and its low contribution in the estimation of only one cluster (coef=-0.05 in cluster 1 [coef. = -0.06 to 1.86]) and null in the other two clusters in LASSO regression(Figure S2, Panel C). Then, final model was calibrated with 227 participants (Figure 2).

Table S1. Association between routine biomarker at baseline and progression of Huntington disease evaluated with cUHDRS*

Routine marker	n	Coef (95%CI)	*p*	*p*-adjusted
Triglycerides	268	-0.37 (-1.27 to 0.52)	0.416	1.000
Triiodothyronine	267	0.97 (0.31 to 1.64)	0.004	0.104
Thyroxine	266	0.18 (-0.01 to 0.36)	0.058	1.000
Thyroid-Stimulating Hormone	275	-0.02 (-0.63 to 0.6)	0.958	1.000
Alanine Aminotransferase	273	0.05 (-0.01 to 0.11)	0.107	1.000
Aspartate Aminotransferase	274	0.06 (-0.03 to 0.15)	0.217	1.000
Gamma-Glutamyl Transferase	269	0.01 (-0.02 to 0.05)	0.503	1.000
Red Blood Cells	274	-0.52 (-1.88 to 0.83)	0.452	1.000
Haemoglobin	274	-0.15 (-0.63 to 0.32)	0.528	1.000
White Blood Cells	274	-0.4 (-0.68 to -0.13)	0.004	0.104
Platelets	272	-0.01 (-0.01 to 0)	0.303	1.000
Sodium	274	0.16 (-0.11 to 0.43)	0.239	1.000
Potassium	273	-0.77 (-2.29 to 0.75)	0.321	1.000
Chloride	269	0.02 (-0.18 to 0.23)	0.818	1.000
Creatinine	274	0 (-0.04 to 0.05)	0.853	1.000
Urea	271	0.56 (0.18 to 0.93)	0.004	0.104
Alkaline Phosphatase	272	-0.01 (-0.04 to 0.01)	0.273	1.000
Total Bilirubin	272	0.06 (-0.06 to 0.18)	0.328	1.000
Neutrophils	274	0.04 (-0.03 to 0.1)	0.241	1.000
Lymphocytes	274	-0.07 (-0.14 to 0.01)	0.083	1.000
Monocytes	271	0.11 (-0.17 to 0.39)	0.456	1.000
High-Density Lipoprotein	264	1.47 (0.14 to 2.81)	0.031	0.806
Total Cholesterol	269	0.34 (-0.21 to 0.88)	0.225	1.000
Blood Glucose	273	0.24 (-0.63 to 1.1)	0.595	1.000
Low-Density Lipoprotein	262	0.65 (0.02 to 1.28)	0.045	1.000
Body Mass Index	324	0.03 (-0.08 to 0.14)	0.614	1.000

**cUHDRS: Composite Unified Huntington's Disease Rating Scale. CAG: cytosine–adenine–guanine. The results of a linear mixed model with cUHDRS as the dependent variable were adjusted for age, age squared, CAG repeats, the interaction of age and CAG repeats, and a random effect at the intercept for each individual. The p-value adjustment was performed using the Bonferroni method.*

Table S2. Population characteristics at first cUHDRS measurement in HD-ISS stage 2 and CAP<150 by routine marker-based profiles.

Characteristics	Cluster 1 (N=95)	Cluster 2 (N=90)	Cluster 3 (N=42)	p value
Age (years)	48.65 (11.10)	48.49 (13.22)	45.93 (12.04)	0.444
Sex				< 0.001
Male	76 (80.0%)	12 (13.3%)	32 (76.2%)	
Female	19 (20.0%)	78 (86.7%)	10 (23.8%)	
Years of education	12.08 (3.57)	12.39 (3.46)	12.86 (3.40)	0.487
CAG repeats	44.22 (3.15)	43.82 (2.72)	44.29 (2.94)	0.574
CAG-Age-Product standardized	102.89 (17.20)	99.14 (17.36)	97.43 (18.00)	0.166
Total motor score	23.77 (16.14)	24.53 (18.99)	19.07 (15.45)	0.219
Symbol Digit Modalities Test	28.20 (12.26)	30.96 (13.95)	34.00 (14.29)	0.057
Stroop word test	69.05 (21.18)	72.03 (21.17)	73.57 (22.25)	0.45
Total Functional Score	11.27 (2.60)	11.50 (2.51)	12.14 (1.39)	0.146
cUHDRS	10.99 (3.97)	11.45 (4.15)	12.50 (3.56)	0.124
Alcohol consumption				0.396
No	89 (93.7%)	88 (97.8%)	40 (95.2%)	
Yes	6 (6.3%)	2 (2.2%)	2 (4.8%)	
Tobacco consumption				0.920
No	67 (70.5%)	65 (72.2%)	31 (73.8%)	
Yes	28 (29.5%)	25 (27.8%)	11 (26.2%)	

*Mean (standard deviation) except where otherwise indicated. *cUHDRS: Composite Unified Huntington's Disease Rating Scale; CAG: cytosine–adenine–guanine. Comparisons performed with analysis of variance and chi-2 test.*

Table S3. Mediating effect of each biomarker on the association between routine marker-based profiles and HD progression

Effect Type	Mediator	Total Effect	Direct Effect	Indirect Effect	Percent Mediated	Total Effect	Direct Effect	Indirect Effect	Percent Mediated	Total Effect	Direct Effect	Indirect Effect	Percent Mediated
Intercept	Alanine Aminotransferase	30.2	28.02	-0.4	1.00%	26.783	25.238	-0.23	1.00%	27.002	26.59	-0.25	1.00%
Slope	Alanine Aminotransferase	-0.78	-0.69	0.00	0.00%	-0.68	-0.62	0.00	0.00%	-0.66	-0.64	0.00	0.00%
Intercept	Alkaline Phosphatase	30.20	28.50	-0.90	3.00%	26.78	26.14	-0.76	3.00%	27.00	26.89	-0.59	2.00%
Slope	Alkaline Phosphatase	-0.78	-0.67	0.00	2.00%	-0.68	-0.61	-0.02	2.00%	-0.66	-0.61	-0.02	3.00%
Intercept	Aspartate Aminotransferase	30.20	28.33	-0.60	2.00%	26.78	25.61	-0.47	2.00%	27.00	27.26	-0.53	2.00%
Slope	Aspartate Aminotransferase	-0.78	-0.70	0.00	0.00%	-0.68	-0.62	0.00	0.00%	-0.66	-0.65	0.00	0.00%
Intercept	Blood Glucose	30.20	26.69	0.90	3.00%	26.78	25.07	0.79	3.00%	27.00	25.28	0.91	3.00%
Slope	Blood Glucose	-0.78	-0.69	0.00	0.00%	-0.68	-0.65	0.00	0.00%	-0.66	-0.63	0.00	0.00%
Intercept	Body Mass Index	30.20	27.96	2.30	8.00%	26.78	25.00	1.86	7.00%	27.00	25.10	1.95	7.00%
Slope	Body Mass Index	-0.78	-0.77	0.00	0.00%	-0.68	-0.68	0.00	0.00%	-0.66	-0.66	0.00	0.00%
Intercept	Creatinine	30.20	27.60	0.00	0.00%	26.78	25.85	-0.03	0.00%	27.00	26.95	-0.04	0.00%
Slope	Creatinine	-0.78	-0.69	0.00	0.00%	-0.68	-0.65	0.00	0.00%	-0.66	-0.66	0.00	0.00%
Intercept	Gamma-Glutamyl Transferase	30.20	27.37	-0.20	1.00%	26.78	25.70	-0.03	0.00%	27.00	27.24	-0.04	0.00%
Slope	Gamma-Glutamyl Transferase	-0.78	-0.68	0.00	0.00%	-0.68	-0.64	0.00	1.00%	-0.66	-0.66	-0.01	1.00%
Intercept	Haemoglobin	30.20	28.02	-0.60	2.00%	26.78	25.85	-0.48	2.00%	27.00	27.10	-0.55	2.00%
Slope	Haemoglobin	-0.78	-0.69	0.00	0.00%	-0.68	-0.63	0.00	0.00%	-0.66	-0.64	0.00	0.00%
Intercept	High-Density Lipoprotein	30.20	25.35	1.30	4.00%	26.78	23.10	1.47	5.00%	27.00	24.58	1.32	5.00%
Slope	High-Density Lipoprotein	-0.78	-0.65	0.00	1.00%	-0.68	-0.60	-0.01	1.00%	-0.66	-0.61	-0.01	1.00%
Intercept	Lymphocytes	30.20	26.97	0.30	1.00%	26.78	24.65	0.33	1.00%	27.00	26.19	0.37	1.00%
Slope	Lymphocytes	-0.78	-0.68	0.00	0.00%	-0.68	-0.62	0.00	0.00%	-0.66	-0.65	0.00	0.00%
Intercept	Neutrophils	30.20	27.59	-0.20	1.00%	26.78	25.46	-0.16	1.00%	27.00	26.61	-0.14	1.00%
Slope	Neutrophils	-0.78	-0.69	0.00	0.00%	-0.68	-0.63	0.00	0.00%	-0.66	-0.64	0.00	0.00%
Intercept	Red Blood Cells	30.20	27.45	0.00	0.00%	26.78	25.40	0.03	0.00%	27.00	26.51	0.04	0.00%
Slope	Red Blood Cells	-0.78	-0.69	0.00	0.00%	-0.68	-0.63	0.00	0.00%	-0.66	-0.64	0.00	0.00%
Intercept	Thyroxine	30.20	26.69	0.40	1.00%	26.78	23.47	0.40	1.00%	27.00	25.57	0.52	2.00%
Slope	Thyroxine	-0.78	-0.67	0.00	0.00%	-0.68	-0.58	0.00	0.00%	-0.66	-0.62	0.00	0.00%
Intercept	Triglycerides	30.20	26.89	-0.20	0.00%	26.78	24.42	-0.09	0.00%	27.00	26.04	-0.04	0.00%
Slope	Triglycerides	-0.78	-0.66	0.00	0.00%	-0.68	-0.59	0.00	0.00%	-0.66	-0.62	0.00	0.00%
Intercept	White Blood Cells	30.20	27.75	-0.30	1.00%	26.78	25.69	-0.26	1.00%	27.00	26.78	-0.19	1.00%
Slope	White Blood Cells	-0.78	-0.69	0.00	0.00%	-0.68	-0.63	0.00	0.00%	-0.66	-0.64	0.00	0.00%

Figure S1. Distribution of all data available of routine markers at base line.

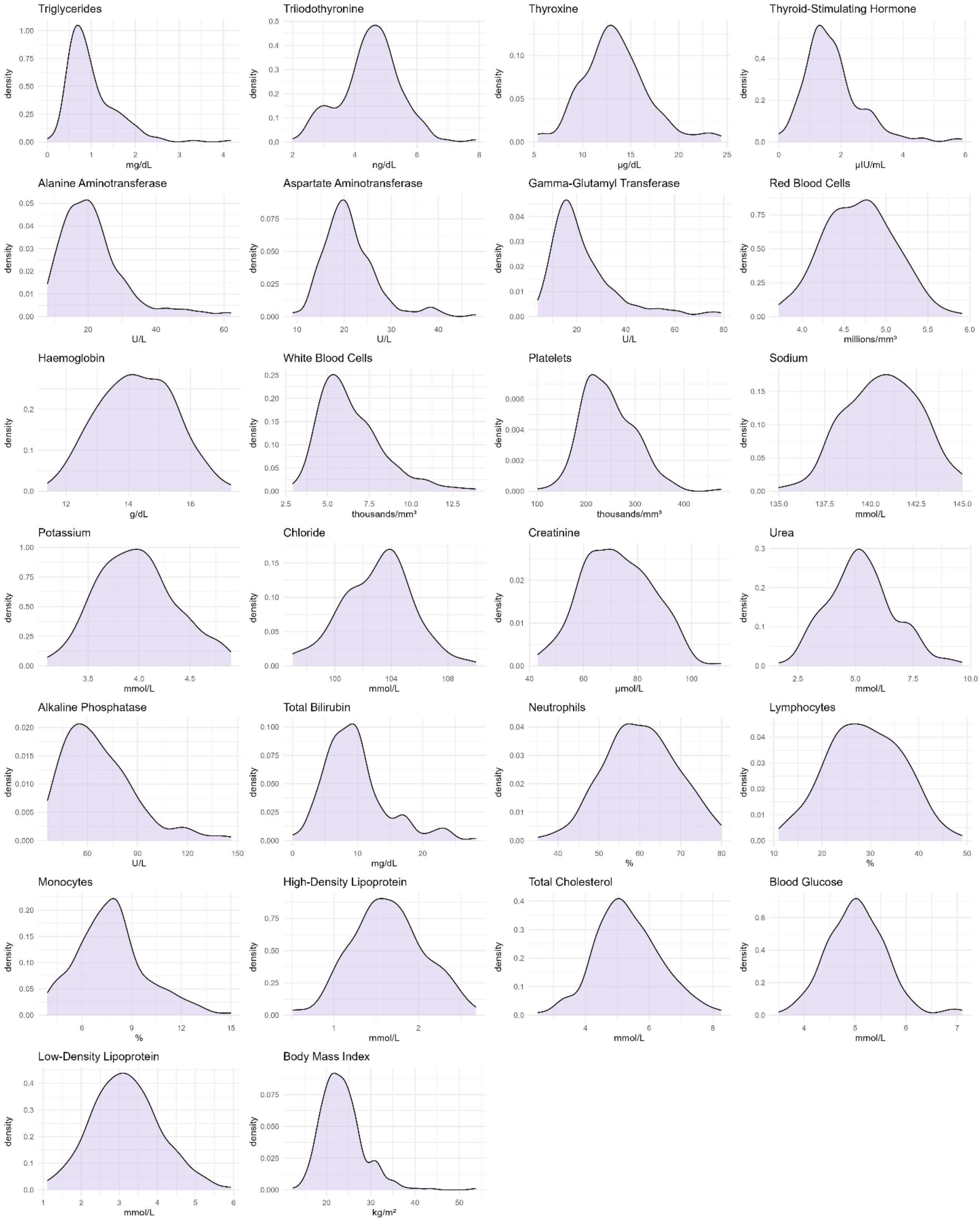

Figure S2. Process of routine biomarker selection.

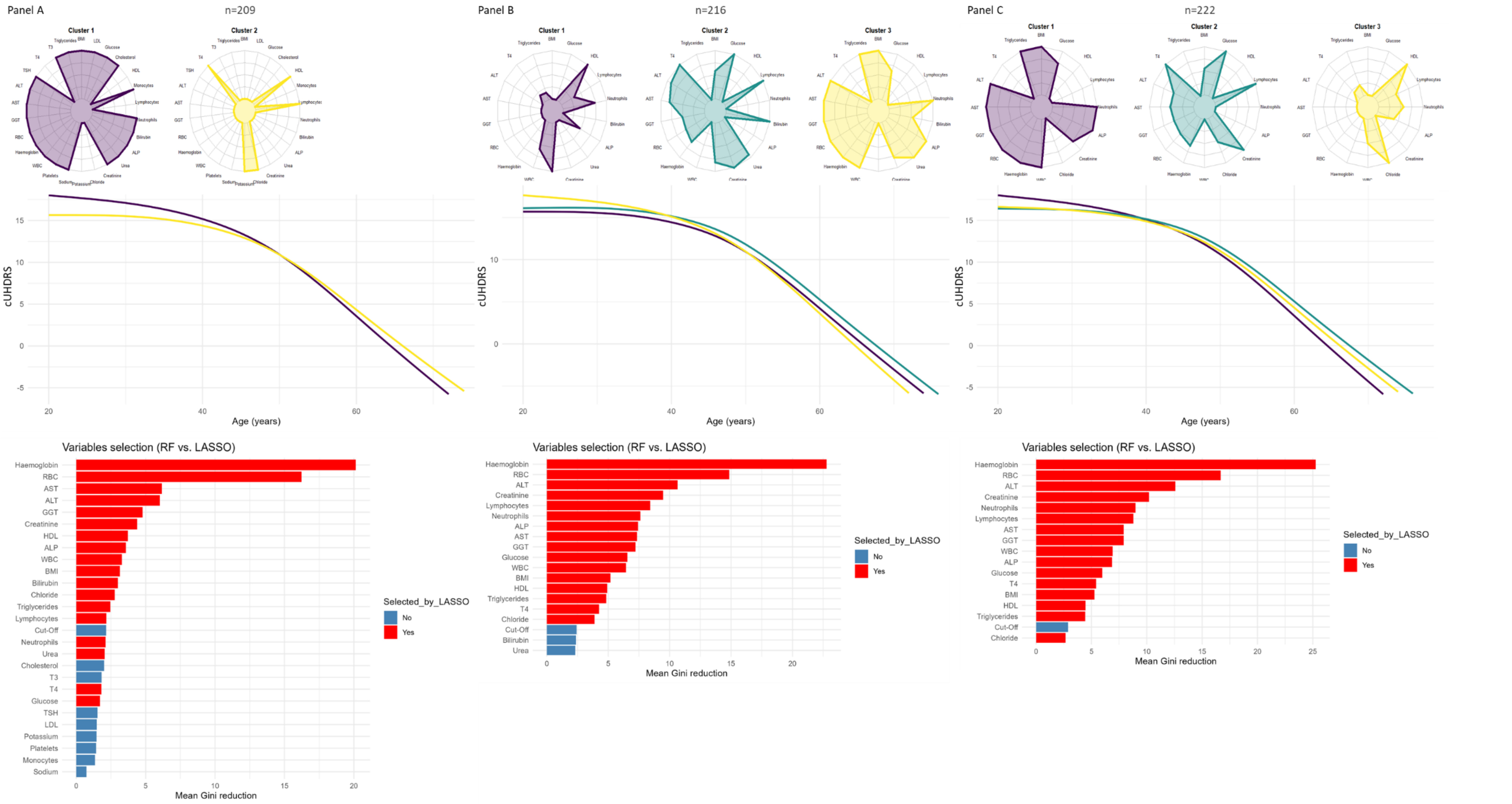

Each panel represents an iteration in the variable selection process. Clusters were estimated using k-means on variables reduced via UMAP. Number of clusters was determined based on differences in cUHDRS trajectories. Trajectories were estimated using a Generalized Additive Model adjusted for age, CAG repeat length, their interaction, and a random effect at the individual level. Variable selection was performed using LASSO regression and a classification Random Forest model. A randomly generated variable was included to define a cut-off for feature importance within the model. Abbreviations: cUHDRS: Composite Unified Huntington's Disease Rating Scale; UMAP: Uniform Manifold Approximation and Projection; GAM: Generalized Additive Model; CAG: Cytosine-Adenine-Guanine; LASSO: Least Absolute Shrinkage and Selection Operator.

Figure S3. UMAP Screening of remaining set of routine biomarkers with K-Means Clustering.

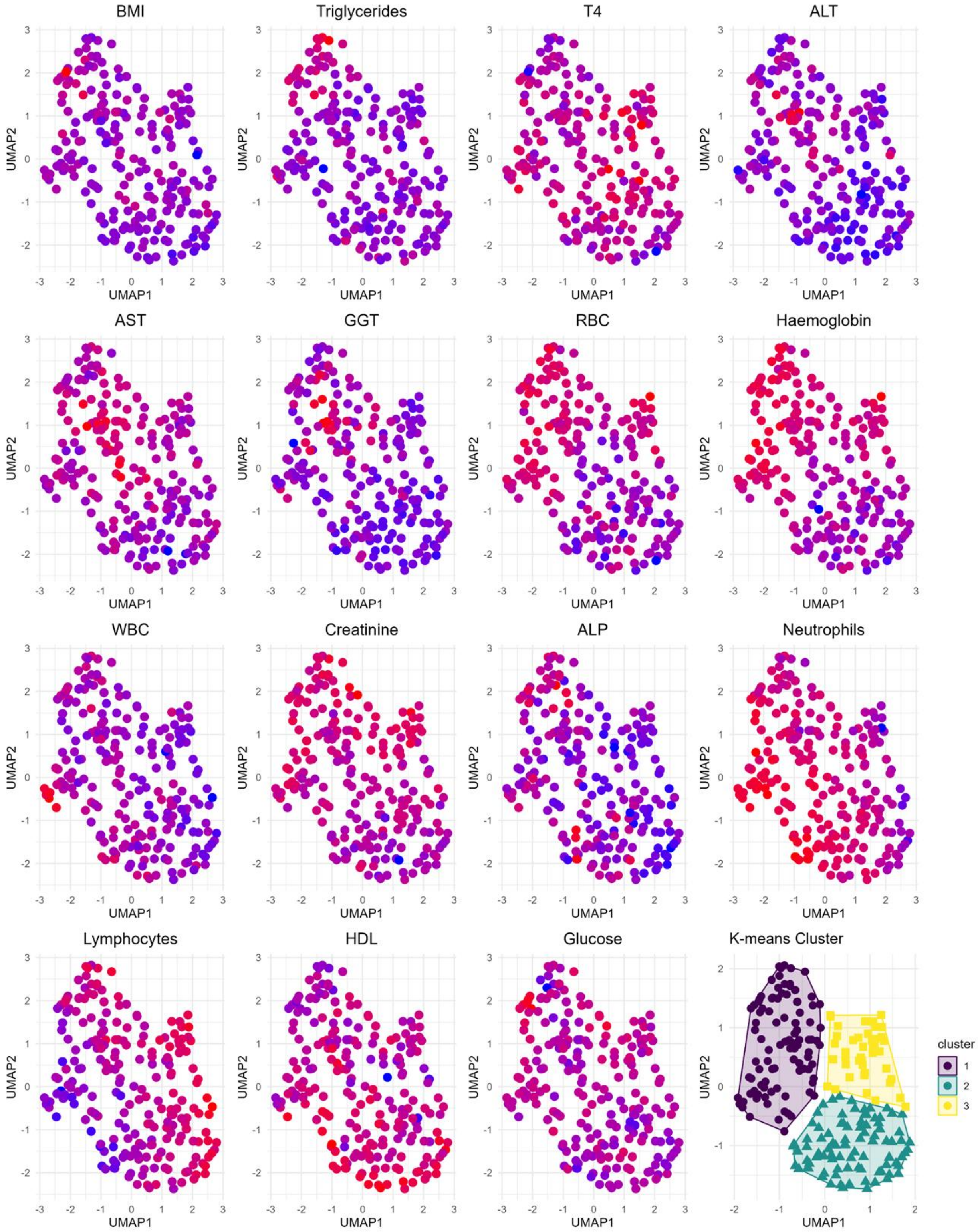

Figure S4. Distribution of remaining set of routine biomarkers by Cluster

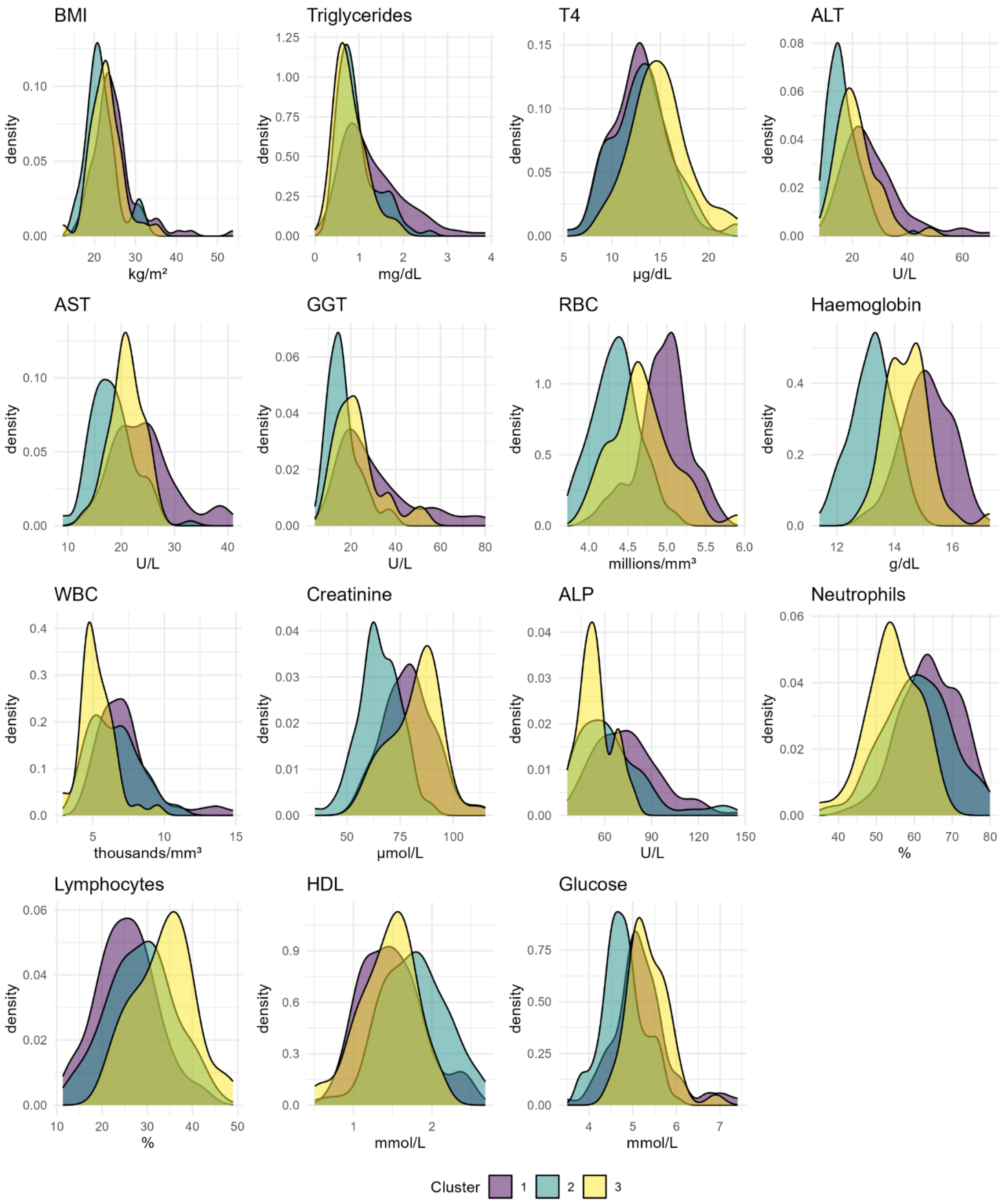

Figure S5. Trajectories of Huntington's disease progression measured with the TMS, SDMT, SWR and TFC as a function of retained -set-based profiles of routine markers at the onset of the manifest phase.

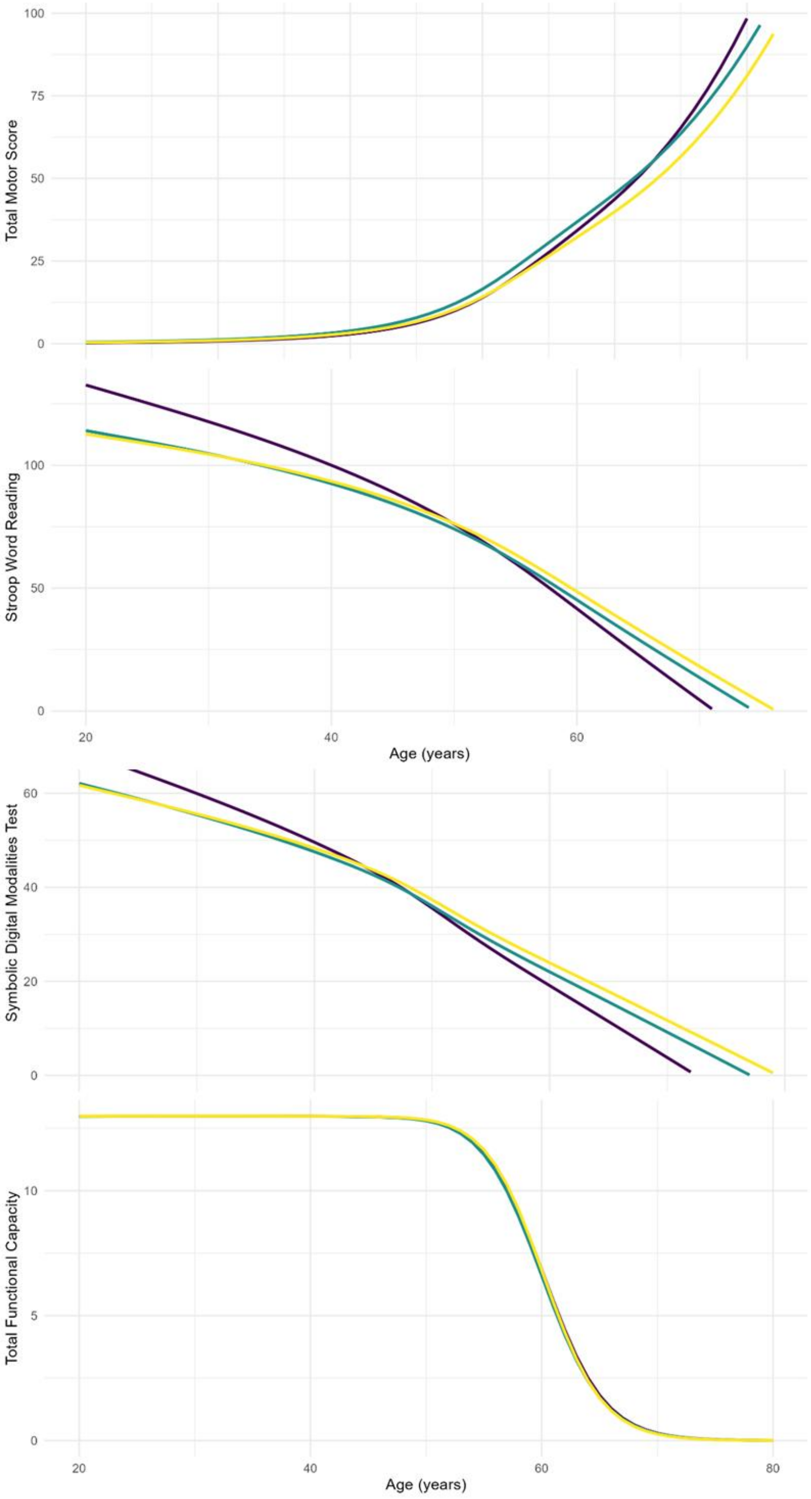

Results of the Generalized Additive Model for TMS, TFC, SDMT and SWR separately with Tweedie, beta and 2 Gaussian distribution respectively. Model was fitted by clusters, years of education, a cubic regression spline with six basis functions for age, a tensor product interaction spline between age and CAG repeats number, a random effect smooth term at the individual level.

Figure S6. **Trajectories of Huntington's disease progression measured with cUHDRS as a function of retained -set-based profiles of routine markers at the onset of the manifest phase with a linear mixed model.**

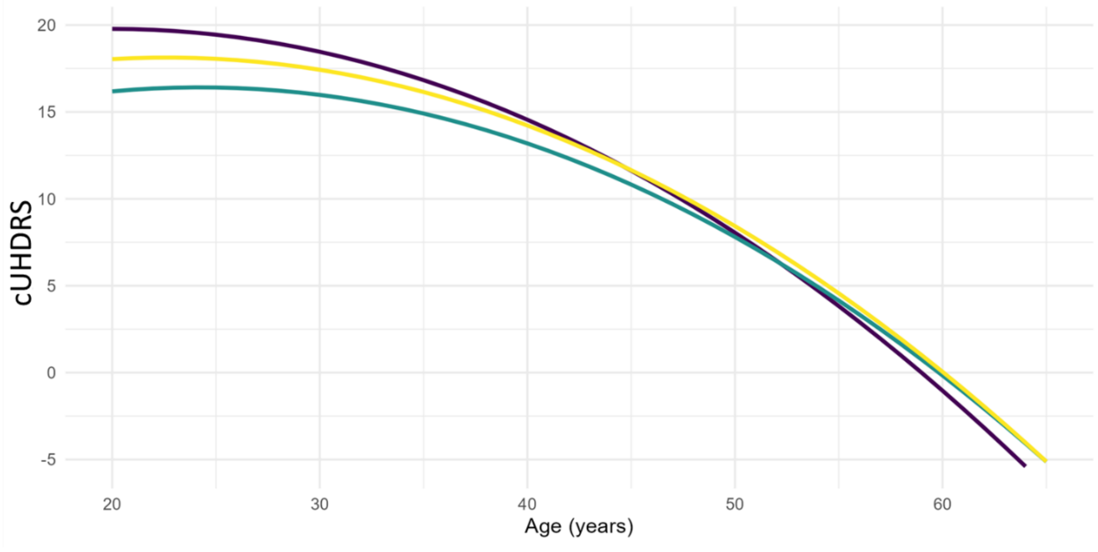

Results of lineal mixed model of cUHDRS in function of clusters and age as time scale. Model was adjusted by years of education, CAG repeats number and interaction between CAG-repeats and age. Random effects at intercept at individual level.

Figure S7. Trajectories of HD progression measured with the cUHDRS as a function of retained-set-based profiles of routine markers in individuals at HD-ISS stage 2, excluding subjects with BMI imputations

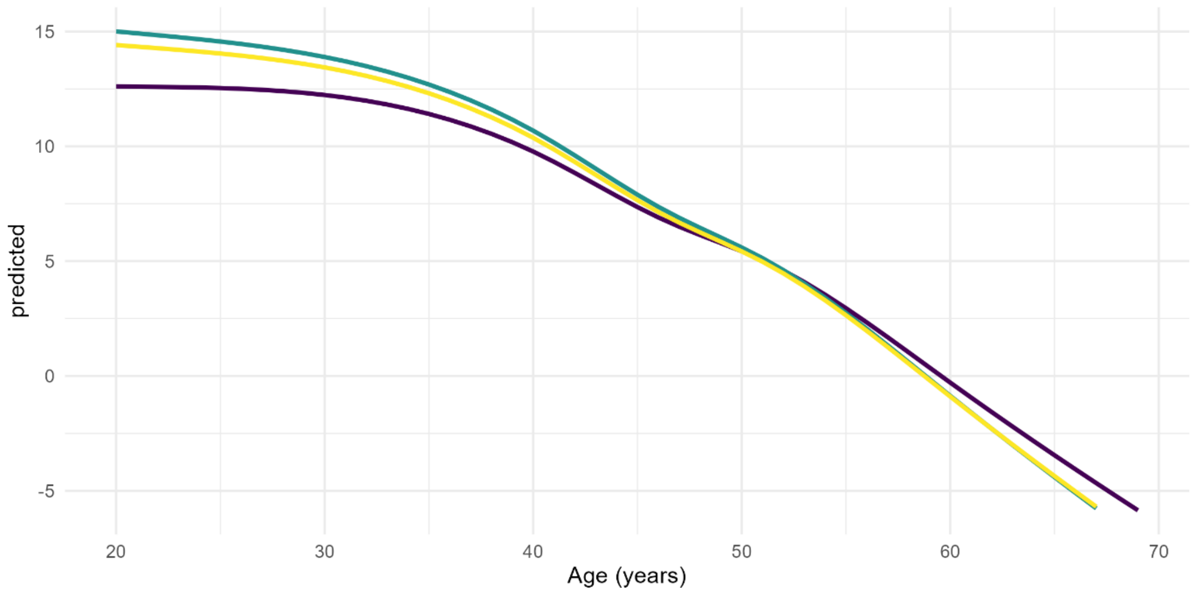

Results of GAM model of cUHDRS in function of clusters and age as time scale. Model was adjusted by years of education, CAG repeats number and interaction between CAG-repeats and age. Random effects at intercept at individual level.
